# Virtual reality-assisted prediction of adult ADHD based on eye tracking, EEG, actigraphy and behavioral indices: a machine learning analysis of independent training and test samples

**DOI:** 10.1038/s41398-024-03217-y

**Published:** 2024-12-31

**Authors:** Annika Wiebe, Benjamin Selaskowski, Martha Paskin, Laura Asché, Julian Pakos, Behrem Aslan, Silke Lux, Alexandra Philipsen, Niclas Braun

**Affiliations:** 1 Department of Psychiatry and Psychotherapy, University Hospital Bonn, Bonn, Germany; 2 Department of Visual and Data-Centric Computing, Zuse Institut Berlin, Berlin, Germany; 3 Department of Clinical Neuroscience, Karolinska Institutet, Stockholm, Sweden

**Keywords:** Diagnostic markers, ADHD

## Abstract

Given the heterogeneous nature of attention-deficit/hyperactivity disorder (ADHD) and the absence of established biomarkers, accurate diagnosis and effective treatment remain a challenge in clinical practice. This study investigates the predictive utility of multimodal data, including eye tracking, EEG, actigraphy, and behavioral indices, in differentiating adults with ADHD from healthy individuals. Using a support vector machine model, we analyzed independent training (*n* = 50) and test (*n* = 36) samples from two clinically controlled studies. In both studies, participants performed an attention task (continuous performance task) in a virtual reality seminar room while encountering virtual distractions. Task performance, head movements, gaze behavior, EEG, and current self-reported inattention, hyperactivity, and impulsivity were simultaneously recorded and used for model training. Our final model based on the optimal number of features (maximal relevance minimal redundancy criterion) achieved a promising classification accuracy of 81% in the independent test set. Notably, the extracted EEG-based features had no significant contribution to this prediction and therefore were not included in the final model. Our results suggest the potential of applying ecologically valid virtual reality environments and integrating different data modalities for enhancing robustness of ADHD diagnosis.

## Introduction

Attention-deficit/hyperactivity disorder (ADHD), characterized by core symptoms of inattention, impulsivity, and hyperactivity [1], is a complex and heterogeneous neurodevelopmental disorder that affects approximately 5.9% of youths and 2.5% of adults worldwide [2]. This heterogeneity is reflected in inter-individual variability in symptom presentation and cognitive deficits, as well as underlying etiological mechanisms [3].

Currently, the diagnostic assessment of adult ADHD symptoms is primarily based on a combination of clinical interviews and self- and informant-rating scales [4, 5]. However, relying on retrospective patient reports is susceptible to biased self-perception and malingering, posing the risk of both false negative and false positive diagnoses [5]. To prevent frequent misdiagnosis [6], it is therefore essential to obtain a maximum number of sources of relevant information, which in turn is time-consuming, costly, and not generally feasible [7].

Further complicating this issue, no single (bio-)marker for ADHD exists to date, although promising candidates have been identified with regard to neuronal activity, physiological parameters, neuropsychological functioning, and molecular-genetics [8–11]. The current lack of reliable physiological or cognitive markers for ADHD can, at least partly, be explained by the above-mentioned high complexity and heterogeneity of the disorder [11]. A potential remedy for this might be combining different categories of markers: For instance, combining a neuropsychological attention test with measures of actigraphy has been shown to improve diagnostic accuracy [12]. Overall, however, the state of research on combining two or more different categories of potential biomarkers is still sparse [9, 10].

In addition, when it comes to neuropsychological tests, the low discriminative ability may also be caused by a lack of ecological validity. The abstract and highly standardized, artificial test environments are unlikely to adequately reflect the complex everyday reality of patients with ADHD [13, 14]. Consequently, the development of assessment methods that capture behavior and physiological responses of affected individuals across multiple measurement modalities in realistic symptom-provoking situations would substantially increase the efficiency of the diagnostic process. Moreover, accurate and situational ADHD symptom tracking is highly relevant for monitoring treatment progress and response to any intervention.

**Fig. 1:**
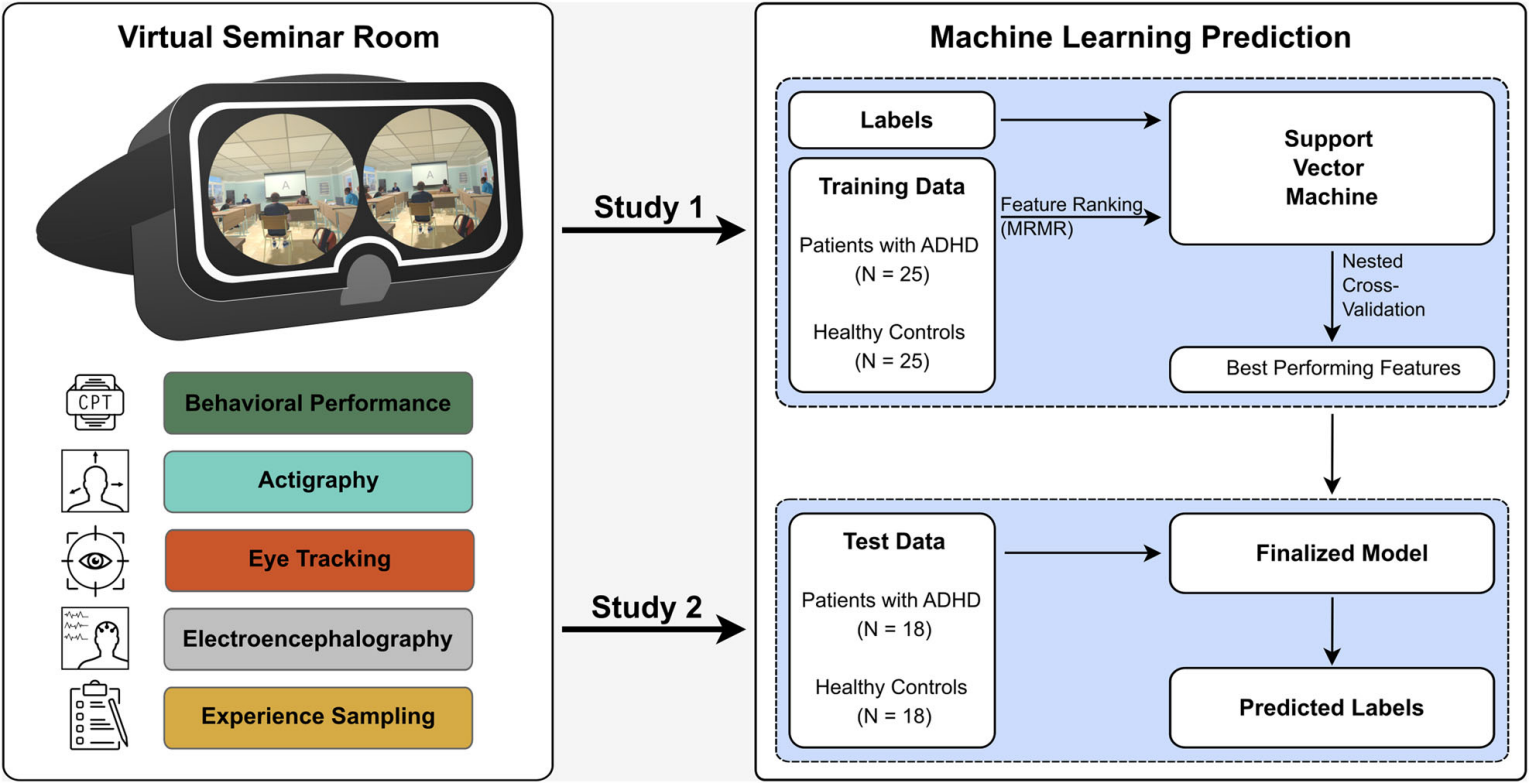
Methodology of the present machine learning analysis. The virtual seminar room (VSR) with multimodal symptom assessment was applied in two different clinical studies. Both samples were used for this analysis, whereby the model was first trained on data from Study 1, and then tested on data from Study 2. MRMR maximal relevance and minimal redundancy.

To improve the situational characterization of ADHD symptoms in adulthood, we have developed a novel, multimodal assessment tool based on virtual reality (VR). Since VR technology can be used to create computerized scenarios with high complexity and realism, ecological validity can be increased while still retaining standardization [15]. As a result, the technology is increasingly being used in a wide range of applications and for a variety of mental disorders [16]. In our developed scenario, which is modeled on existing virtual classroom scenarios for children with ADHD [17, 18], participants are immersed in a virtual seminar room (VSR) where they complete a continuous performance task (CPT, originally developed by Rosvold et al. [19]) while distracting events occur [20]. Simultaneously, to characterize ADHD symptomatology multimodally, several measurement domains are combined: CPT performance, head actigraphy, eye tracking, electroencephalography (EEG), and experience sampling (see Fig. 1).

The VSR has now undergone several clinical trials, including an assessment to differentiate between medicated and unmedicated patients with ADHD as well as healthy controls (HC) [21], in the context of an eye tracking-based attention refocusing training [22], and in combination with transcranial alternating current stimulation [23]. Similar group differences were found in several domains in the two former studies, in which both patients and HC participated: attention performance (CPT), head movements (actigraphy), gaze behavior (eye tracking), and experience sampling [21, 22]. Yet, although evidence of group differences provides an important initial indication of the scenario’s discriminative capacity, it still has limited diagnostic value. Therefore, there is a need to examine the extent to which the combination of the different domains in the VSR enables classification of individuals with and without ADHD at single-subject level and to determine which of the different domains are most informative.

Machine learning can support such analyses and is becoming increasingly valuable in the investigation of mental disorders such as ADHD. Previous application included ADHD subgroup clustering [24, 25], medication response prediction (84.6% accuracy) [26], classification of individuals with ADHD and HC using a large neuroimaging dataset (0.62 area under the curve, AUC) [27], and differentiating children with and without ADHD in a clinical sample with an accuracy of 66.1%, using symptom ratings, age, gender, and neuropsychological measures [28]. Another study aimed to classify patients with ADHD, with obesity, with problematic gambling, and HC based exclusively on the items of a self-assessment of ADHD symptoms (Conners’ Adult ADHD Rating Scale), age, and gender, and found a global accuracy of 80% [29]. The ADHD diagnosis for study participation, however, was determined via an ADHD interview, in which items with similar content were rated within the same study inclusion procedure, suggesting a high correlation between the two metrics. Although a recent review identified research gaps with respect to clinical decision support for all modalities of ADHD assessments except magnetic resonance imaging [30], there are some promising predictive classification models for children with ADHD that have included other measures such as features of a VR attention task, achieving an accuracy of 83.2% [31], or used eye movement data obtained during a visual search task in VR, yielding high discriminant ability (0.92 AUC) [32].

However, all these studies used models of low generalizability without including robust validation processes such as independent validation sets [33]. A recent review investigating the generalizability of clinical prediction models in schizophrenia found that while the models generally demonstrated high accuracy within the respective trials, out-of-sample application often failed to exceed chance level [34]. This finding highlights the risk of overestimating model performance that has not been tested in independent samples [35]. In addition, contrary to our VSR assessment most previous studies seem to lack an integration of multiple concurrent symptom measures, although a multifactorial causation of ADHD is assumed and a multimodal assessment approach allows for improved detection of interindividual variability [3].

Our primary research questions revolve around the utility of combining these different domains for classifying individuals with ADHD and HC. Specifically, we aim to answer the following questions:

1. Can our multimodal VSR assessment significantly differentiate between adults with ADHD and HC based on a machine learning analysis of independent training and test sets?
2. To what extent can the integration of multiple measurement domains, including CPT performance, head actigraphy, eye tracking, EEG, and experience sampling, enhance the accuracy of differentiating individuals with ADHD and HC?
3. Which of the measurement domains contribute most significantly to the discriminative power of the classification model?

To address these research questions, we analyzed data from two clinical studies that assessed adult patients with ADHD and HC in the VSR. For the machine learning-based analysis, a model was trained on a dataset consisting of 25 patients with ADHD and 25 HC [21], which was then tested on an independent test sample of 18 patients with ADHD and 18 HC [22] (see Fig. 1).

## Methods

Both studies were conducted in accordance with the Helsinki Declaration as revised in 2013, approved by the University of Bonn’s medical ethics committee (protocol numbers: 113/21 and 297/20) and preregistered in the German Clinical Trials Register (https://www.drks.de/, trial-IDs: DRKS00025824, DRKS00022370). Recruitment for the training sample was conducted between September 2021 and July 2022; recruitment for the test sample was conducted between February 2021 and August 2021. Participants with ADHD were mainly recruited via the specialist outpatient clinic for adult ADHD of the Department for Psychiatry and Psychotherapy of the University Hospital Bonn, and, to a small proportion, via publicly accessible media. HC were recruited via publicly accessible media. Both studies were carried out in the Department for Psychiatry and Psychotherapy of the University Hospital Bonn. The present analysis is reported in accordance with the transparent reporting of a multivariable prediction model for individual prognosis or diagnosis (TRIPOD) checklist for prediction model development and validation [36] and recently published set of guidelines for reporting machine learning investigations in neuropsychiatry (GREMLIN) [37].

### Participants

Participants in the ADHD group were required to meet diagnostic criteria for ADHD as outlined in the Diagnostic and Statistical Manual of Mental Disorders (DSM-5) [1]. This was ensured through a two-step process. First, participants had to either provide proof of an official ADHD diagnosis from a specialist or had to be patients at the specialist outpatient clinic for adult ADHD at the University Hospital Bonn which entails a detailed diagnostic process including self- and informant-assessment. Second, as part of the study procedure, the diagnosis was verified via a structured clinical interview. In addition, considering that ADHD has high comorbidity rates, the presence of comorbid mental disorders was assessed (for details, see General experimental procedure). As we aimed to ensure a high level of generalizability of the results, occurrence of comorbidities was not a reason for exclusion per se. However, because the investigation focused on core ADHD symptoms, severe mental or neurological disorders, which may mask the characteristics of ADHD, were excluded. Thus, individuals were ineligible to participate if they met diagnostic criteria for schizophrenia spectrum disorders, severe affective disorders, (moderate to severe) substance use disorder, epilepsy, or other significant neurological disorders. In the training set, individuals with severe anxiety disorders, tic disorders, and severe obsessive-compulsive disorder were additionally excluded. For safety reasons, pregnancy was an exclusion criterion as well. HC were ineligible if they met the diagnostic criteria for ADHD or any of the other exclusion criteria as described above. All eligible participants gave written informed consent.

It should be noted that there were differences between the training and test sets with respect to medication status. Specifically, the training set comprised participants with ADHD not currently under medication, while the test set involved participants who had discontinued their ADHD medication for a 48-h interval preceding the experimental phase. Based on the maximum duration of action of common ADHD medications of about 13 h, the expected impact on the current analysis is assumed to be minor [38].

### General experimental procedure

The general experimental procedure was similar in both experiments and consisted of two separate appointments for each participant. On the first appointment, all participants underwent an extensive diagnostic assessment. First, a DSM-5-based, structured clinical interview on ADHD symptoms (IDA-R [39]) was carried out to confirm the (suspected) diagnosis for participants in the ADHD group and to rule out ADHD symptomatology for HC participants. Beyond assessing current ADHD symptoms in adulthood, this includes an evaluation of ADHD symptoms before the age of 12. If, based on the interview, a participant in the ADHD group did not meet the required diagnostic criteria, or if an HC participant met the criteria for ADHD, they were withdrawn from the study. In addition, a second structured clinical interview on common mental disorders based on DSM-5 criteria (MINI-DIPS [40]) was administered to rule out any mental disorder-related exclusion criteria and to assess potential comorbidities. Further, participants filled out a battery of online questionnaires via SoSci Survey (https://www.soscisurvey.de/), including questionnaires on demographics (self-developed), self-rated ADHD symptoms (ADHS-SB [41]), depression, anxiety, and stress symptoms (DASS [42]), and quality of life (WHO-QOL BREF, German version [43, 44]).

On the second appointment, the actual VR experiment was carried out in the VR laboratory of the psychiatry department of the University Hospital Bonn. Participants immersed into the VSR (see Fig. 1) via the head-mounted display HTC VIVE Pro Eye (HTC Corporation, Taoyuan City, Taiwan). The VSR was created in Unity 3D (Version: 2019.1.10f1, Unity Technologies, San Francisco, CA, USA) and C#. In the VSR, participants underwent a virtual CPT. The task consisted of single letters appearing consecutively on the canvas in the front of the virtual room, with a stimulus interval of 100 ms and an inter-stimulus interval of 1100 ms. The letter sequence A-K ( ~ 30% of all letter pairs) was the target sequence which required participants to press the spacebar on a keyboard in front of them, while all other letter sequences ( ~ 70% of all letter pairs) were non-targets. Half of the non-targets were pseudo-target sequences, in which the first letter was ‘A’, but the second not ‘K’, or the second letter was ‘K’, but the first not ‘A’. In total, participants went through three CPT blocks of 18 min (450 letter pairs) each, whereby each block was subdivided into alternating 3-min distractor (DP) and non-distractor phases (NDP). In a DP, every 30 s a different distracting stimulus (visual, auditory, or audio-visual) was played in the VSR. Based on participants’ key presses, CPT performance parameters, such as error rates, mean response times, and response time variability were calculated for both DP and NDP (for details, see Supplementary Table 1). After each block, a gesture-controlled user interface was displayed by means of which participants expressed their momentary level of inattention, impulsivity, and hyperactivity on a Likert scale from −3 to 3. At the end of the experiment, the same user interface was used to ask participants about their experienced realness, user satisfaction, and virtual reality sickness (Virtual Reality Sickness Questionnaire [45]).

In the dataset used as test set, participants additionally received an eye tracking-based visual and auditory feedback signal test during the task. In each block, a different one of three feedback conditions (real eye tracking feedback, sham feedback, no feedback) was presented, whereby their order was counterbalanced between participants. Thus, for each participant, one of the experimental blocks contained no feedback signal, rendering this block identical to the training set. Only this block without feedback was used for the present secondary analysis. For detailed information on the eye tracking-based attention training, see Selaskowski et al. [22].

While individuals performed the virtual CPT, their head movements, eye movements, and EEG were recorded simultaneously (further details in the respective sections below). Further, in the training set, brain activity was recorded via functional near-infrared spectroscopy, which, however, is not part of this secondary analysis. All data streams were recorded and synchronized via Lab Streaming Layer (LSL; https://github.com/sccn/labstreaminglayer). Preprocessing and calculation of all parameters used as features for the machine learning analysis was done in Matlab 2021b (The MathWorks Inc., Natick, MA, USA). Afterwards, the machine learning analysis was carried out in Python 3.11 (Python Software Foundation, https://www.python.org/).

### Head actigraphy

Head movements were captured via the 3D position and rotation tracking of the HTC Vive with a sampling rate of ~90 Hz. For offline analyses, data were first down-sampled to ~10 Hz and for each 3D coordinate the Euclidean distance to the preceding coordinate was calculated, for both head position and rotation. Next, the mean Euclidean distance for head position and rotation across all respecting samples was computed for each DP and NDP. Finally, these values were averaged across all DP and across all NDP to obtain the average changes in head position and head rotation for DP and for NDP.

### Eye tracking

Eye movements were recorded with an infrared-based Tobii eye tracker (Tobii Technology, Stockholm, Sweden) built into the head-mounted display. The eye tracker has an estimated accuracy of 0.5° to 1.1° and data was sampled with a frequency of ~50 Hz. Eye tracking data were collected using a combination of software packages, including SRanipal SDK, Tobii XR SDK, and Lab Streaming Layer (for a detailed description of the eye tracking setup and data processing, see Wiebe et al. [21], Selaskowski et al. [22]). This software composition enabled the recording of participants’ gaze focus on various objects within the virtual environment, in particular on the canvas used for the presentation of the CPT. Gaze behavior was thereby categorized into three states, which could not occur simultaneously: task focus, distractor focus, and gaze wandering.

For offline analysis, eye tracking data were processed using Matlab. A wide range of parameters based on saccades, fixations, and blinks were calculated and used as features for the present machine learning model. The detection algorithm was based on a custom script which has been described in detail [22].

### EEG

EEG data were recorded with a wireless, Bluetooth-based EEG system (Smarting^®^, mBrainTrain^®^, Belgrade, Serbia) with a 500 Hz sampling rate and 24-bit step-size resolution. An EEG cap (EASYCAP, Herrsching, Germany) was equipped with 24 Ag/AgCL sintered ring electrodes, using the following positions of the 10–20 system: Fp1, Fp2, AFz, F3, Fz, F4, T7, C3, Cz, C4, T8, CPz, P7, P3, Pz, P4, P8, POz, O1, O2, M1, and M2. The ground electrode (DRL) was placed at FPz and the reference electrode (CMS) at FCz. For offline analyses, the Matlab toolbox EEGLAB 2021.0/2021.1 was used. Following preprocessing (for detailed information, see Wiebe et al. [21], Selaskowski et al. [22]), for the frequency analysis, each DP and NDP for each block was cut into non-overlapping, 5-s segments. Next, for each artifact-free segment, a continuous wavelet transformation was conducted for channels Fz, Cz, and Pz (time resolution: 0.004 s, frequency range: 0.10-35 Hz in 85 steps (log scale)). Then, across all DP and across all NDP per block, the average theta (4–7 Hz), alpha (8–12 Hz), and beta (13–30 Hz) power across all frequency and time bins within 0.5–4.5 s was calculated. Lastly, for the training set, the mean powers for DP and for NDP across all three blocks were computed.

### Machine learning pipeline

A total of 76 parameters were calculated from the recorded measurement domains CPT performance, head actigraphy, eye tracking, EEG, and experience sampling as described above and used as features for the machine learning analysis (for the complete list of features, see Supplementary Table 1). All available features were included in the initial model without any pre-selection or aggregation. The data tables containing all features of interest were transferred to Python. For machine learning preprocessing, model development, and classification, the package scikit-learn [46] was used.

First, missing data was imputed using the k-nearest neighbor algorithm (*k* = 10). Only in the training set, EEG data for one patient with ADHD and one HC, and eye tracking data (except gaze behavior) for two patients with ADHD were missing completely and therefore imputed. In one further patient with ADHD and one HC, data were only missing in single blocks (for details, see Wiebe et al. [21]). For these two participants, the total averages of all features were calculated across the remaining two blocks, without imputation. After missing data handling, in both training and test set all features were rescaled using min-max normalization.

A linear support vector machine (SVM) [47] was chosen as the classification algorithm, because SVMs are capable of processing high-dimensional data and are well suited for binary classification problems [48], such as distinguishing between individuals with ADHD and HC. SVMs aim to find an optimal hyperplane that maximizes the spread between the two classes, providing an effective means of detecting patterns in complex datasets. Due to their good balance between accuracy and generalizability, even in small samples [49], SVM are among the most widely employed algorithms in diagnostic and biomarker research in psychiatry [50, 51].

Using only the training set, the SVM was fitted, and relevant features were selected. Model performance was thereby evaluated via nested cross-validation (CV), which, compared to regular CV, has been shown to better prevent overfitting in small samples [52]. In nested CV, model hyperparameter tuning is carried out in an inner loop that is nested inside the CV loop for model selection [53]. In our case, grid search was used to find the optimal value of the regularization parameter C (grid: [0.1, 1, 10, 100]). For the inner loop, a three-fold split, and for the outer CV loop, a five-fold train/test split was applied. The overall CV model performance score was then obtained by calculating the average performance score across all five folds.

To determine the optimal number of features, all available features were first ranked using the maximal relevance and minimal redundancy (MRMR) algorithm based on the MRMR python package (https://github.com/smazzanti/mrmr, version 0.2.8). The MRMR algorithm, which was first described by Ding and Peng [54], ranks the features by having minimal similarity (e.g. minimal pairwise correlations) with each other while still being highly correlated to the class variable.

Next, a model with all available features was trained and evaluated using nested CV. Subsequently, based on the MRMR ranked features, individual features were successively removed, starting with the lowest ranked feature. From this, the minimum required number of features that yielded the best nested CV performance scores was identified. Using the same feature ranking, the models were then trained on the entire training set and their performance evaluated in the independent test set.

Model performance was assessed via three metrics: the overall ratio of correctly classified cases (accuracy), the ratio of correctly identified positive cases, i.e. patients with ADHD (sensitivity), and the ratio of correctly identified negative cases, i.e. HC (specificity).

Further, the statistically significant threshold for classification accuracy was calculated using a binomial cumulative distribution, following the formula suggested by Combrisson and Jerbi [55].

## Results

Detailed clinical characteristics and demographic information of the two samples used for training and test data are presented in Table 1.

**Table 1:** Demographic information and clinical characterization of training and test samples.

Characteristic	Training set, No. (%)		Test set, No. (%)	
	ADHD (*n* = 25)	HC (*n* = 25)	ADHD (*n* = 18)	HC (*n* = 18)
Mean Age (SD)	31.28 (9.5)	31.04 (8.7)	36.1 (10.7)	25.9 (3.1)
Age Range	20-50	18-50	22-62	23-34
Gender^a^				
Male	15 (60.0)	15 (60.0)	12 (66.6)	11 (61.1)
Female	10 (40.0)	10 (40.0)	6 (33.3)	7 (38.9)
Education				
≤ Intermediate certificate	3 (12)	6 (24)	6 (33.3)	0
Higher education entrance degree	13 (52)	10 (40)	6 (33.3)	9 (50.0)
Higher education degrees	9 (36)	9 (36)	6 (33.3)	9 (50.0)
ADHD presentation^b^				
Predominantly inattentive	8 (32)	–	7 (38.9)	–
Predominantly hyperactive-impulsive	2 (8)	–	0	–
Combined	15 (60)	–	11 (61.1)	–
ADHD symptom severity, mean (SD)^b^	36.8 (5.9)	9.6 (6.5)	33.6 (7.3)	7.4 (5.5)
Inattention, mean (SD)	20.9 (3.9)	4.8 (3.0)	18.8 (3.1)	4.8 (3.6)
Impulsivity, mean (SD)	7.6 (2.2)	2.9 (1.6)	6.9 (2.6)	1.2 (1.8)
Hyperactivity, mean (SD)	8.3 (3.7)	2.0 (2.1)	7.8 (3.6)	1.3 (1.8)
Current comorbid disorders^c^				
Affective disorders	1 (4)	0	0	0
Anxiety disorders	4 (16)	1 (4)	8 (44.4)	2 (11.1)
Other disorders (OCD, PTSD, sleep disorders, Gaming/Gambling disorder)	9 (36)	1 (4)	2 (11.1)	0
Depression symptoms, mean (SD)^d^	12.28 (4.7)	7.84 (1.3)	10.1 (1.7)	8.8 (4.2)
Anxiety symptoms, mean (SD)^d^	10.40 (3.9)	8.04 (1.7)	10.6 (2.7)	8.6 (2.5)
Quality of life total score, mean (SD)^e^	62.9 (14.8)	80.7 (13.0)	59.6 (11.8)	80.6 (13.6)

*ADHD* attention-deficit/hyperactivity disorder, *OCD* obsessive-compulsive disorder, *PTSD* post-traumatic stress disorder, *SD* standard deviation.

^a^None of the participants identified as non-binary.

^b^According to observer rating via the Integrated Diagnosis of ADHD in Adulthood (IDA-R [39]).

^c^According to the Diagnostic Brief Interview for Mental Disorders (MINI-DIPS [40]).

^d^According to the Depression Anxiety Stress Scale (DASS [42]).

^e^According to the World Health Organization Qualify of Life Questionnaire (brief version, WHOQOL-BREF [43, 44]), mean across subscales.

### Classification

**Fig. 2:**
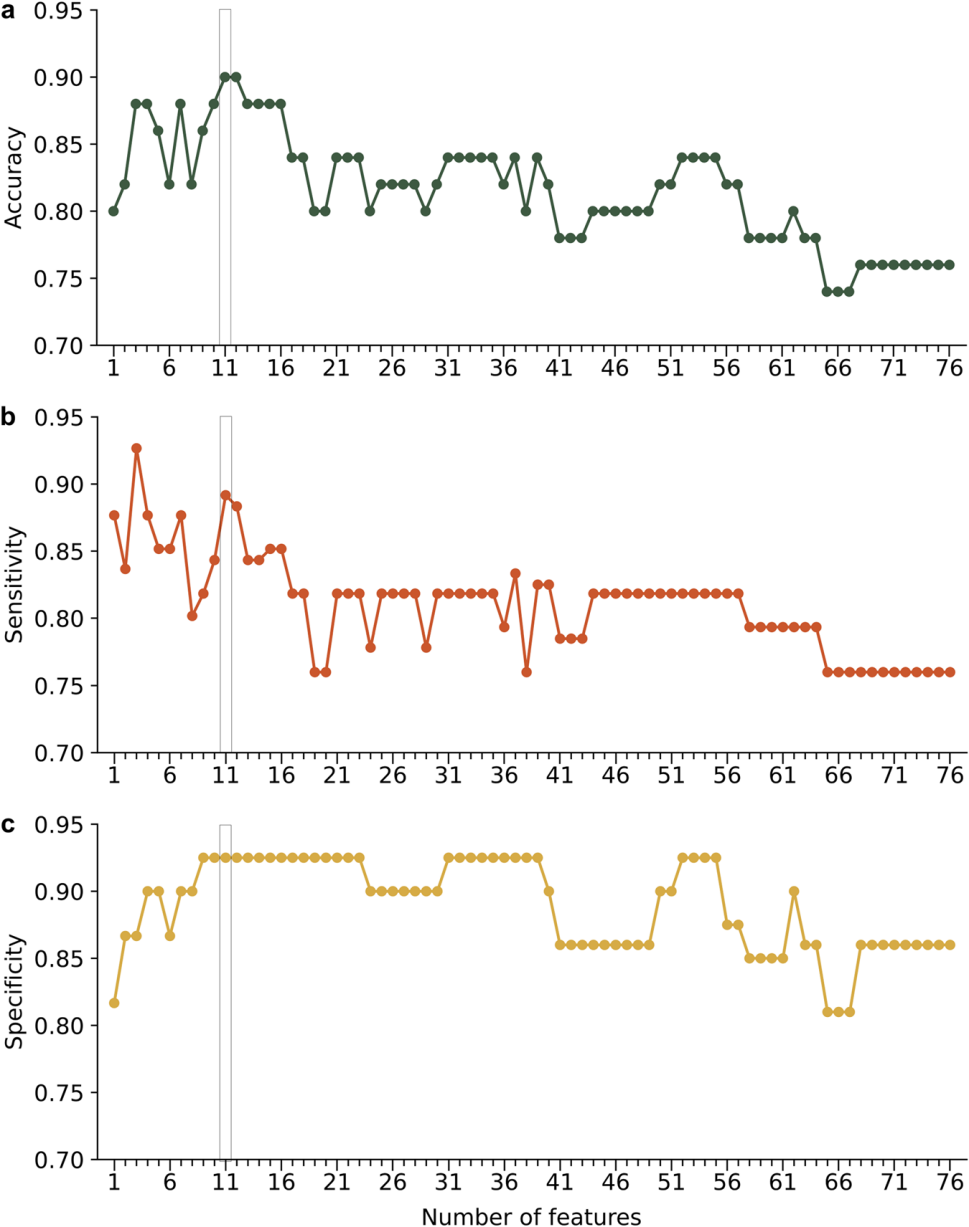
Performance of trained support vector machine model based on nested cross validation within the multimodal training data. Classification **a** accuracy, **b** sensitivity, and **c** specificity as a function of number of features ranked by the maximal relevance and minimal redundancy criterion. As indicated by the rectangle, 11 is the number of features with highest accuracy.

When considering all 76 extracted features, the linear SVM yielded a CV accuracy of 0.76, a sensitivity of 0.76, and a specificity of 0.86 in the training set. Nested CV performance as a function of the number of features, ranked by MRMR criterion, can be found in Fig. 2. This indicates that the highest CV accuracy (0.90), while still maintaining high sensitivity (0.89) and specificity (0.93), can be achieved with 11 features.

**Fig. 3:**
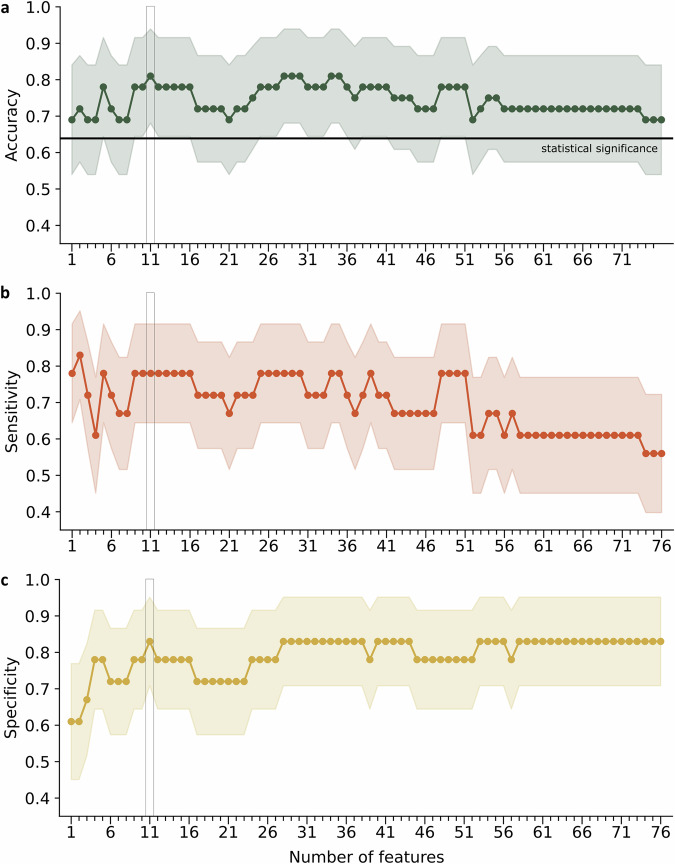
Performance of trained support vector machine model in the independent test set. Classification **a** accuracy, **b** sensitivity, and **c** specificity as a function of number of features ranked by the maximal relevance and minimal redundancy criterion. The black horizontal line represents the statistical significance threshold. Shaded areas illustrate the 95% confidence intervals. As indicated by the rectangle, the 11 features that performed best in the training set equally show the highest possible accuracy in the test set.

Training the model with all 76 extracted features on the whole training set and evaluating its performance in the independent test set yielded a test accuracy of 0.69 (confidence interval [CI]: 0.54, 0.84), a sensitivity of 0.56 (CI: 0.40, 0.72), and a specificity of 0.83 (CI: 0.71, 0.95). Test accuracy, sensitivity, and specificity as a function of the number of features can be found in Fig. 3. Again, the 11 highest ranked features from the training set proved to be robust as the minimum required features with highest accuracy. Here, test accuracy was 0.81 (CI: 0.68, 0.94), exceeding the statistically significant threshold of 0.64. Sensitivity amounted to 0.78 (CI: 0.64, 0.92), and the specificity to 0.83 (CI: 0.71, 0.95).

**Fig. 4:**
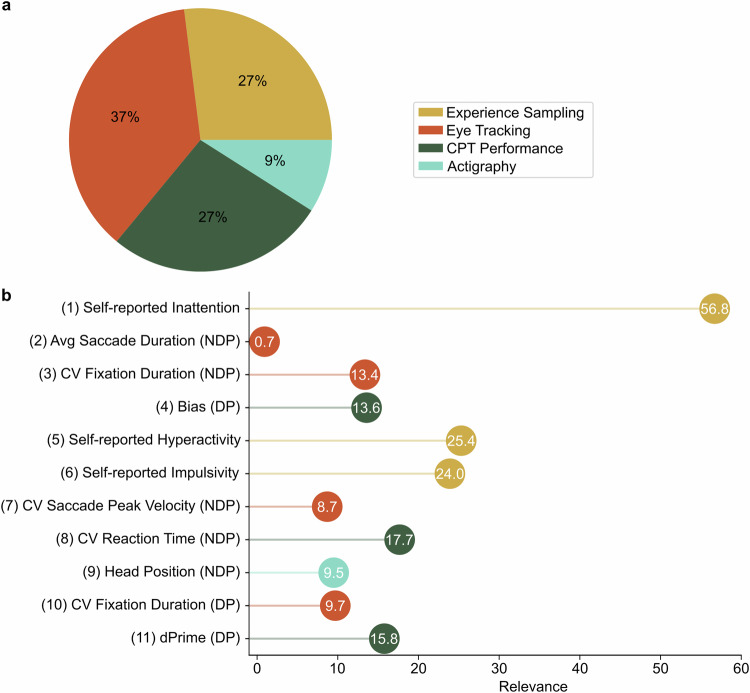
Domain contribution and relevance of the 11 best performing features. **a** Relative contribution of the five domains to the 11 best performing features. **b** Relevance of 11 best performing features (in order of ranking) and corresponding domains indicated by colors. Avg Average, CV Coefficient of variation, CPT Continuous performance task, DP Distractor phases, NDP Non-distractor phases.

For the final model including 11 features, varying relevance of the different measurement domains for the discrimination between patients with ADHD and HC was found. The model achieving the most accurate classification is composed of three experience sampling features, four eye tracking features, three CPT features, and one head actigraphy feature, while EEG did not contribute with any parameter. A schematic representation of the domain proportions as well as a list of the individual parameters is shown in Fig. 4.

The final 11 features included in the model can be described as follows. Self-reported inattention, hyperactivity, and impulsivity are user queries derived from experience sampling during VSR performance. Here, questions were asked at certain points in time within the VSR, in which participants gave a self-assessment regarding their respective ADHD core symptomatology via a gesture-controlled interface (for a detailed description and sample questions, see Wiebe et al. [20]). Additionally, four eye tracking parameters are included, three of which were recorded during phases without additional virtual distractors (NDP): average saccade duration across all saccades in the recorded block without distractors; coefficient of variation of fixation durations, which is a measure of the variability of a variable via the ratio of standard deviation to mean, in this case reflecting the variability of the recorded fixation durations; and coefficient of variation of saccade peak velocity, which describes the variability in the maximum velocity of the individual saccades made; lastly, as the only eye tracking measure during phases of additionally recorded virtual distractors (DP), coefficient of variation of fixation duration is included in the model. Three CPT task parameters were incorporated, including two from DP, namely bias and dPrime (for details on the exact calculation, see Supplementary Table 1). Bias is the tendency of the respondent to answer too little or too much in relation to the actual occurrence of the respective target stimuli [56]. dPrime describes the ability of the participant to distinguish between signal (hit rate) and noise (false alarm rate), calculated as a standard score [56]. The coefficient of variation of reaction time was further added to the model from the CPT during NDP. Finally, the change in head position during NDP was integrated as the only parameter from the actigraphy modality.

## Discussion

The primary research question of the present investigation was to determine how accurately our multimodal VSR can differentiate between individuals with ADHD and HC. Using 76 features based on five measurement domains (CPT, head actigraphy, eye tracking, EEG, experience sampling), the linear SVM yielded a CV accuracy of 0.76 in the training set and an accuracy of 0.69 in the independent test set. Reducing the number of features according to the MRMR criterion revealed a maximum accuracy of 0.90 (CV) and 0.81 (independent test set) based on 11 features.

Previous classification studies of individuals with ADHD and HC yielded accuracies in a similar range. Yeh et al. [31], for example, used CPT performance and head actigraphy measures in a virtual classroom and were able to differentiate between children with ADHD from HC (6-12 years old) with an accuracy of up to 83.2%. Likewise, investigating CPT performance measures in 6–12 year old children, Slobodin et al. [57] achieved an accuracy of 87%, a sensitivity of 89%, and a specificity of 84% in the classification of a large ADHD sample. An SVM trained only on eye tracking data in a VR task by Merzon et al. [32] revealed an AUC of 0.92, sensitivity of 0.84, and specificity of 0.78 in a sample of 9-13 year old children. Investigating the predictive value of EEG frequency power in an adult sample, Tenev et al. [58] found a maximum accuracy of 82.3%. However, all above-mentioned studies lacked an independent test set, and most studies did not investigate adult samples which hinders comparability with the present study. Considering the frequent overestimation of model performance by CV [35], the present finding of a comparable accuracy in an independent test set indicates superiority of our model for future clinical application. This may point towards an advantage of combining multiple feature domains within one diagnostic model. Moreover, by using an independent test set that utilized different experimenters, measurement times, and general procedures on the study day, as well as differences in sample characteristics compared to the training set, the VSR suggests reliability even under heterogeneous conditions in clinical practice.

Further, with respect to the research questions on the influence of a multimodal combination of different domains on the classification accuracy as well as the specific domains that appear relevant for adult ADHD, the following conclusions emerge. The 11 best-performing features all originated from experience sampling, gaze behavior, attention performance, and head movements (for details, see Methods section), while none were derived from the EEG domain. Consequently, an additional measurement of EEG power does not seem to provide additional discriminatory value. This corresponds to previous findings regarding EEG frequency bands as markers for adult ADHD, which have so far been inconsistent [59]. However, it should be noted that alternative EEG parameters (e.g., event-related potentials, ERPs) may potentially be more relevant for classification. For instance, a study by Kim et al. [60] demonstrated promising results for classifying ADHD by using features derived from the ERP mismatch negativity. Overall, all domains except EEG are represented in the final model, demonstrating that a combination of a variety of potential markers could provide superior coverage of heterogeneous ADHD symptoms than previous attempts to detect a single (bio-)marker.

Notably, although the VSR was developed with the aim of decreasing the influence of self-report in ADHD symptom assessment, three important features of the machine learning classification are based on participants’ self-assessment. This type of assessment appears to have limitations, given the impaired self-awareness of patients with ADHD regarding their own symptoms and cognitive performance. Studies have found that self-report scales indicate more severe cognitive impairment than performance tests [61] and that informant-reports on ADHD symptoms may be more accurate than self-reports [62]. Hence, there is a possibility that the VSR assessment may add limited value due to the high proportion of self-report features and the resulting similarity to common questionnaire approaches. However, the use of experience sampling presented here counteracts one of the potentially significant disadvantages of traditional diagnostics, namely the bias introduced by retrospective symptom evaluation. Whereas previous correlational findings have shown substantial associations between VSR experience sampling-based symptom assessment and retrospective observer- as well as self-report symptom ratings in healthy individuals, no such correlations were found in patients with ADHD [22]. Experience sampling may reflect a different dimension than previous retrospective questionnaire assessments in ADHD. In line with this, Butzbach et al. [63] found that while individuals with ADHD reported deficits in self-awareness, they rated their performance in a neuropsychological attention test with similar accuracy as a healthy control group. Thus, one aim of future research is to determine whether the integration of the presented type of momentary self-assessment should become a stronger diagnostic focus for ADHD in adults.

### Limitations and future directions

The main limitation of the present study is the relatively small sample size (*n* = 50 in the training set; *n* = 36 in the independent test set). However, our use of an independent test set provided external validation of our results and thus significantly reduced a major weakness of small sample sizes in machine learning, namely the risk of overfitting and resulting lack of generalizability [35, 64].

Still, as a result of our small sample size, subgroup analysis of different ADHD symptom presentations was not feasible. Yet, machine learning-based differentiation of core symptom profiles could offer a more personalized treatment approach, especially given some indication that core symptoms respond differently to treatment attempts such as first-line medication [65]. Beyond ADHD symptoms, the approach might also be relevant with regard to etiological subgroups and precisely tracking treatment progress [66]. A few studies already pointed towards an advantage of this dimensional approach. In Tenev et al.’s [58] EEG study, for example, the discrimination accuracy between ADHD presentations was higher than that of the global differentiation between individuals with ADHD and HC. Moreover, Fair et al. [67] found that clustering children with ADHD and HC based on neuropsychological test profiles indicated an increase in classification accuracies compared to categorization only based on clinical diagnosis. A multimodal approach, in which the clustering is supplemented by additional feature domains, e.g., eye tracking and actigraphy, may provide even more precise information on subgroups and contribute to greater accuracy. Consequently, the VSR should be applied to a significantly larger study sample to increase the generalizability of the results.

The above-mentioned potential benefit of neuropsychological clustering is further supported by previous studies investigating combined samples of children with ADHD, HC, and/or individuals with borderline personality disorder [68] or autism [25] who trained classifiers to discriminate transdiagnostic groups. Future VSR studies should therefore additionally focus on expanding the sample to patients with comorbidities and other patient groups, especially regarding disorders with high symptom overlap, such as borderline personality disorder or autism. Mikolas et al. [28] used machine learning to differentiate children and adolescents with ADHD (*n* = 299) from a wide range of other mental disorders using anonymized data from clinical records, including symptom rating scales and neuropsychological parameters, and achieved a CV accuracy of 66.1%. Based on their findings, which suggested importance of a relatively broad combination of symptoms across domains for a possible ADHD diagnosis, it was concluded that research efforts aimed at identifying physiological markers for ADHD that rely less on (informant-reported) symptom ratings need to be continued, and that multimodal data specifically could improve this detection. In this context, an important focus for future research should be to directly compare performance of a classification model trained on the multimodal VSR data with a model trained on routinely assessed clinical data such as self- and informant-symptom ratings. This would provide quantifiable information on whether the multimodal approach has a clinically relevant diagnostic benefit, in particular considering the specific challenges in ADHD diagnostics such as heterogeneous presentation or transdiagnostic differentiation.

If, as the present results indicate, EEG features do not have added informational value for classification beyond the other domains, it may be omitted in the VSR paradigm. While recording of eye tracking, actigraphy, and experience sampling during a CPT block requires no additional administration time, except for the approximately two-minute calibration of the eye tracker, EEG is both more time-consuming and more costly to implement. In addition, the analysis of EEG data in particular is considerably more time-consuming compared to analyses within the other modalities. Omitting EEG measurement could reduce the total assessment duration of the VSR from more than 60 min to about 30 min, including preparation time. Moreover, acquisition and operational costs associated with EEG could be avoided. Likewise, a routine clinical implementation of the VSR could become feasible and possibly substitute neuropsychological computerized testing of attention parameters in the future. Its clinical relevance, on the other hand, must be evaluated in a multicentric study providing large-scaled independent training and test samples to draw reliable conclusions.

In our study, validation in an independent test set has already been implemented. In particular, given that a recent review found that high within-sample prediction accuracy of trained models may in many cases not exceed chance level when applied out-of-sample [34], we provide substantial evidence for the robustness of our findings. Yet, an evaluation of our trained model in a new sample recorded by a completely independent research group would provide additional evidence for the robustness of our results.

## Conclusion

In conclusion, the present analysis provides robust evidence that the VSR can reliably differentiate between adults with ADHD and healthy individuals, as emphasized by the employment of an independent test set. Our results underscore the utility of employing a multidimensional approach in the assessment of ADHD symptoms, by demonstrating that the combination of attention performance, head movements, gaze behavior, and experience sampling data enhances diagnostic accuracy. EEG had no added predictive value beyond these domains. Overall, the multimodal VSR emerges as a promising avenue for advancing our ability to precisely characterize, diagnose, and track treatment progress in individuals with ADHD.

## Supplementary information

Supplementary Table 1

## Data Availability

Dataset, variable coding list, and underlying code supporting the conclusions of this work are available in the Open Science Framework (OSF) repository, https://osf.io/ewkgv/ (10.17605/OSF.IO/EWKGV).
