# Supplementary material for: Virtual reality-assisted prediction of adult ADHD based on eye tracking, EEG, actigraphy and behavioral indices: a machine learning analysis of independent training and test samples
Source: Transl Psychiatry. 2024 Dec 31;14:508. doi: 10.1038/s41398-024-03217-y (PMC11688437; doi:10.1038/s41398-024-03217-y)
Supplement: Supplementary file 1 — Supplementary Table 1 [file 41398_2024_3217_MOESM1_ESM.pdf]

Supplementary Table 1

1. CPT Performance

Variable Code	Meaning
CPT_DP_Mean_RT_Hits	Mean reaction time of correct hits in distractor phases
CPT_DP_CV_Hits	Reaction time variability (coefficient of variation; CV) of correct hits in distractor phases<br>CV = standard deviation/mean
CPT_DP_FalseAlarmRate	Commission error rate in distractor phases
CPT_DP_MissRate	Omission error rate in distractor phases
CPT_DP_SA_Tradeoff	Speed/accuracy-trade-off in distractor phases<br>SA-trade-off = mean reaction time/hit rate
CPT_DP_bias	Response bias (signal detection parameter) in distractor phases<br>bias = -(z(hit rate) + z(commission error rate)/2
CPT_DP_dPrime	d-prime (signal detection parameter) in distractor phases<br>d' = z(hit rate)-z(commission error rate)
CPT_NDP_Mean_RT_Hits	Mean reaction time of correct hits in non-distractor phases
CPT_NDP_CV_Hits	Reaction time variability (coefficient of variation; CV) of correct hits in non-distractor phases<br>CV = standard deviation/mean
CPT_NDP_FalseAlarmRate	Commission error rate in non-distractor phases
CPT_NDP_MissRate	Omission error rate in non-distractor phases
CPT_NDP_SA_Tradeoff	Speed/accuracy-trade-off in non-distractor phases<br>SA-trade-off = mean reaction time/hit rate
CPT_NDP_bias	Response bias (signal detection parameter) in non-distractor phases<br>bias = -(z(hit rate) + z(commission error rate)/2
CPT_NDP_dPrime	d-prime (signal detection parameter) in non-distractor phases<br>d' = z(hit rate)-z(commission error rate)

2. Head Actigraphy

Variable Code	Meaning
Acti_DP_Movement	Head position changes in distractor phases
Acti_DP_Rotation	Head rotation in distractor phases
Acti_NDP_Movement	Head position changes in non-distractor phases
Acti_NDP_Rotation	Head rotation in non-distractor phases

3. Eye Tracking

Variable Code	Meaning
ET_DP_Attending_Distractors	Mean time spent looking at distractors in distractor phases
ET_DP_Attending_Canvas	Mean time spent looking at canvas in distractor phases
ET_DP_Mindwandering	Mean time spent gaze wandering (looking away from canvas but not looking at a distractor) in distractor phases
ET_DP_Distractability_Score	Mean distractibility score ((time of distractor focus + time of gaze wandering) / time of canvas focus) in distractor phases

ET_DP_Num_Saccades	Mean number of saccades in distractor phases
ET_DP_Avg_Dur_Saccades	Mean duration of saccades in distractor phases
ET_DP_CV_Dur_Saccades	Duration variability of saccades in distractor phases
ET_DP_Avg_Vel_Saccades	Mean velocity of saccades in distractor phases
ET_DP_CV_Vel_Saccades	Velocity variability of saccades in distractor phases
ET_DP_Peak_Vel_Saccades	Highest velocity of saccades in distractor phases
ET_DP_CV_Peak_Vel_Saccades	Variability of highest velocity of saccades in distractor phases
ET_DP_Num_Fixations	Mean number of fixations in distractor phases
ET_DP_Avg_Dur_Fixations	Mean duration of fixations in distractor phases
ET_DP_CV_Dur_Fixations	Duration variability of fixations in distractor phases
ET_DP_Avg_Vel_Fixations	Mean velocity of fixations in distractor phases
ET_DP_Num_Blinks	Mean number of blinks in distractor phases
ET_DP_Avg_Dur_Blinks	Mean duration of blinks in distractor phases
ET_DP_CV_Dur_Blinks	Duration variability of blinks in distractor phases
ET_NDP_Attending_Canvas	Mean time spent looking at canvas in non-distractor phases
ET_NDP_Mindwandering	Mean time spent gaze wandering (looking away from canvas but not looking at a distractor) in non-distractor phases
ET_NDP_Distractability_Score	Mean distractibility score ((time of distractor focus + time of gaze wandering) / time of canvas focus) in non-distractor phases
ET_NDP_Num_Saccades	Mean number of saccades in non-distractor phases
ET_NDP_Avg_Dur_Saccades	Mean duration of saccades in non-distractor phases
ET_NDP_CV_Dur_Saccades	Duration variability of saccades in non-distractor phases
ET_NDP_Avg_Vel_Saccades	Mean velocity of saccades in non-distractor phases
ET_NDP_CV_Vel_Saccades	Velocity variability of saccades in non-distractor phases
ET_NDP_Peak_Vel_Saccades	Highest velocity of saccades in non-distractor phases
ET_NDP_CV_Peak_Vel_Saccades	Variability of highest velocity of saccades in non-distractor phases
ET_NDP_Num_Fixations	Mean number of fixations in non-distractor phases
ET_NDP_Avg_Dur_Fixations	Mean duration of fixations in non-distractor phases
ET_NDP_CV_Dur_Fixations	Duration variability of fixations in non-distractor phases
ET_NDP_Avg_Vel_Fixations	Mean velocity of fixations in non-distractor phases
ET_NDP_Num_Blinks	Mean number of blinks in non-distractor phases
ET_NDP_Avg_Dur_Blinks	Mean duration of blinks in non-distractor phases
ET_NDP_CV_Dur_Blinks	Duration variability of blinks in non-distractor phases

4. EEG

Variable Code	Meaning
EEG_DP_Theta_Fz	Mean theta power on Fz electrode in distractor phases
EEG_DP_Theta_Pz	Mean theta power on Pz electrode in distractor phases
EEG_DP_Theta_Cz	Mean theta power on Cz electrode in distractor phases
EEG_DP_Alpha_Fz	Mean alpha power on Fz electrode in distractor phases
EEG_DP_Alpha_Pz	Mean alpha power on Pz electrode in distractor phases
EEG_DP_Alpha_Cz	Mean alpha power on Cz electrode in distractor phases
EEG_DP_Beta_Fz	Mean beta power on Fz electrode in distractor phases
EEG_DP_Beta_Pz	Mean beta power on Pz electrode in distractor phases
EEG_DP_Beta_Cz	Mean beta power on Cz electrode in distractor phases
EEG_NDP_Theta_Fz	Mean theta power on Fz electrode in non-distractor phases
EEG_NDP_Theta_Pz	Mean theta power on Pz electrode in non-distractor phases

EEG_NDP_Theta_Cz	Mean theta power on Cz electrode in non-distractor phases
EEG_NDP_Alpha_Fz	Mean alpha power on Fz electrode in non-distractor phases
EEG_NDP_Alpha_Pz	Mean alpha power on Pz electrode in non-distractor phases
EEG_NDP_Alpha_Cz	Mean alpha power on Cz electrode in non-distractor phases
EEG_NDP_Beta_Fz	Mean beta power on Fz electrode in non-distractor phases
EEG_NDP_Beta_Pz	Mean beta power on Pz electrode in non-distractor phases
EEG_NDP_Beta_Cz	Mean beta power on Cz electrode in non-distractor phases

5. Experience Sampling

Variable Code	Meaning
ES_Impulsivity	Mean self-reported impulsivity score during the experiment
ES_Hyperactivity	Mean self-reported hyperactivity score during the experiment
ES_Inattention	Mean self-reported inattention score during the experiment
ES_Sickness	Mean self-reported sickness score during the experiment
ES_Realness	Mean self-reported perception of realness of the experiment
